# Molecular and phenotypic characterization of 5-FU resistant colorectal cancer cells: toward enrichment of cancer stem cells

**DOI:** 10.1186/s12935-025-03758-2

**Published:** 2025-04-18

**Authors:** Amirhesam Babajnai, Saeed Rahmani, Mohammad Jamal Asadi, Elmira Gheytanchi, Glavizh Adibhesami, Faezeh Vakhshiteh, Zahra Madjd

**Affiliations:** 1 Oncopathology Research Center, Iran University of Medical Sciences, Tehran, Iran; 2 Department of Computer Engineering, Sharif University of Technology, Tehran, Iran; 3 Department of Molecular Medicine, Faculty of Advanced Technologies in Medicine, Iran University of Medical Sciences, Tehran, Iran

**Keywords:** Cancer stem cell, Colorectal cancer, 5-FU, EMT, ABC, Drug resistance

## Abstract

Cancer stem cells (CSCs) as a subgroup of cells within a tumor capable of self-renewal, thereby driving tumor initiation and spread. Addressing treatment failures in cancer, linked to CSCs and their resistance mechanisms, requires effective preclinical models for testing targeted therapies. Caco2- and HT-29-resistant cells were generated by repeated treatment of cells with growing concentrations of 5-fluorouracil (5-FU) anticancer drug for an extended time. The sensitivity of 5-FU-resistant cells was evaluated by cytotoxicity assay. Stemness, epithelial-mesenchymal transition (EMT), migration and drug resistance characteristics were assessed through gene expression investigation by real-time PCR. The expression of CD44, CD133, and CD66 were evaluated by flow cytometry. To end, the bioinformatic analysis estimated the molecular function and biological pathways considering the differential expression of selected genes and proteins. 5-FU-exposed cells displayed increased resistance to 5-FU. The gene expression analysis showed an upregulation of stemness genes (*KLF4*,* SOX2*,* OCT4*,* C-MYC*), enhanced scavenging system, and elevated expression of CSC surface markers (CD44 and CD133) compared to parental cells. Additionally, pro-EMT genes (*TWIST1*,* SNAIL1*,* ZEB1*,* Vimentin*,* and N-cadherin*) were significantly upregulated compared to parental cells, with the downregulation of *E-cadherin* as an EMT suppressor gene reflected in increased migration capacity. Moreover, increased expression of ABC transporter genes (*ABCB1*,* ABCC1*) was observed, correlating with enhanced drug resistance. The bioinformatic analysis highlighted pathways related to microRNAs in cancer, cells pluripotency, and proteoglycans. Methods of drug exposure take priority over spheroid formation, particularly due to their enhanced efficacy in stemness, EMT, and surface markers. This positions them as a promising protocol for establishing experimental models of CSCs.

## Introduction

Colorectal cancer (CRC) is one of the most prevalent malignancies globally and stands as the foremost reason for cancer-related mortality [1]. The estimated number of new colorectal cancer cases in the United States for both sexes is 152,810, with an anticipated 53,010 resulting deaths [2]. Despite advancements in therapeutic approaches such as surgery, chemotherapy, and radiotherapy, which have notably enhanced patient survival rates, the recurrence and spread of the disease remain primary contributors to CRC-related fatalities [3]. High CRC mortality correlates with conventional chemo/radiotherapy resistance, which results in metastasis, tumor recurrence, and aggressive behavior [4–6]. Several mechanisms have been investigated to elucidate resistance to anticancer therapies, such as cell survival signaling pathways, stemness, cancer cell metabolism, antiapoptotic mechanisms, and cellular phenotypes [7].

An accumulating body of evidence suggests that resistance to conventional treatment modalities stems from a subgroup of cells known as cancer stem cells (CSCs) [6, 8–10]. CSCs represent a subset of malignant cells possessing stem cell-like attributes, including the capacity for self-renewal and differentiation across multiple cell lineages [11]. Many challenges in cancer treatment, including treatment resistance, aggressive tumor behavior, recurrence, spread to other parts of the body, and poor patient outcomes, are closely tied to the characteristics of CSCs. These characteristics primarily involve their ability to undergo epithelial-to-mesenchymal transition (EMT), activate signaling pathways related to stemness and pluripotency, and exhibit heightened ATP-binding cassette (ABC) transporter genes expression [12, 13]. Thus, achieving complete tumor regression necessitates a comprehensive insight into of the biological traits of CSCs to explore innovative strategies for targeting this specific cell population [14].

Given the complexities associated with identifying and targeting CSCs within the tumor microenvironment (TME) and understanding their unique biological features, it is imperative to invest efforts in exploring innovative techniques for isolating CSC properties [15]. Various methodologies have been employed to separate, define, and enrich CSCs across diverse cancer types, including using surface markers, sphere formation assays, clonogenic growth assays, and assessments of drug resistance [16–19]. However, the complexity of CSC biology necessitates the continual creation of viable and relevant models for enriching CSC populations.

Typical chemotherapy drugs aim to induce apoptosis in rapidly dividing cells. While effective cancer treatment eliminates much of the actively proliferating tumor cells, a portion of CSCs often survive and contribute to resistance to chemotherapy. The unique feature of “Chemoresistance” can be a feasible target for isolating CSCs from heterogeneous cancer cell populations [20, 21]. Prolonged exposure to chemotherapy drugs may initially reduce the bulk of tumor cells, including non-stem cancer cells. However, some evidence suggests that CSCs might exhibit heightened resistance to chemotherapy in contrast to other cancerous cells which may lead to CSC enrichment in tumor [22].

Understanding the molecular characteristics of CSCs is crucial for developing effective therapeutic strategies. Several methods have been employed to enrich CSC-like populations, including spheroid formation assays and drug selection approaches [18]. Spheroid culture allows CSCs to proliferate in non-adherent, serum-free conditions, selecting for pre-existing stem-like cells [18]. Alternatively, prolonged chemotherapy exposure provides a clinically relevant model that selects for drug-resistant CSC populations, mimicking tumor adaptation during treatment [23].

In this study, we aimed to compare two CSC enrichment strategies: (1) long-term exposure to increasing concentrations of 5-FU, the standard chemotherapeutic agent for CRC [24], and (2) spheroid formation under non-adherent conditions. We evaluated the biological and molecular characteristics of CSC-like populations derived from these approaches by assessing stemness markers, EMT-related genes, and drug resistance-associated proteins. Additionally, we investigated ROS levels to explore the antioxidant defense mechanisms associated with CSC-like traits. By comparing these two enrichment methods, this study provides insights into CSC biology and highlights potential strategies for improving CRC treatment outcomes.

## Materials

Basic fibroblast growth factor (bFGF) (PeproTech, USA), high glucose DMEM (Gibco, Germany), 3-(4,5-Dimethylthiazol-2-yl)-2,5-diphenyltetrazolium bromide (MTT) (Gibco, Germany), fetal bovine serum (FBS), B27 supplement (Gibco, Germany), trypsin/EDTA (Gibco, Germany), cDNA synthesis kit (YektaTajhiz, Iran), DMEM/F12 (Gibco, Germany), epidermal growth factor (EGF) (PeproTech, USA), TRIzol (Sigma-Aldrich, USA), Nanodrop (Biotek, USA), Rotor-Gene Q LightCycler (Qiagene, Germany), CD44, CD133, and CD166 (Abcam, USA), anti-rabbit IgG-FITC (1:100) (Santa Cruz Biotechnology, USA), L-glutamine (Gibco, Germany), Carboxyfluorescein succinimidyl ester (CFSE) Cell Division Tracker Kit (BioLegend^®^, USA), ROS kit (Kiazist^®^, Iran).

## Methods

### Culture conditions and cell lines

The HT-29 and Caco2 CRC cell lines (Iranian Biological Resource Center). These cells were cultured in high glucose DMEM, 10% FBS, and L-glutamine. Culture condition was in a conventional incubator set at 37 °C with a gas mixture consisting of 5% CO2 and 95% humidity to provide a standard cell culture environment. The process of passaging was carried out once the cells achieved a confluence of 70–90%.

### 5-FU sensitivity of HT-29 and Caco2 cell lines

The sensitivity of HT-29 and Caco2 cell lines to 5-FU was assessed by MTT cytotoxicity assay. Initially, 10,000 cells/well were cultured in 100 µl/well of culture medium. After 24 h, the cells were exposed to serial dilutions of 5-FU (6.25, 12.5, 25, 50, 100, 200, 400, 800, 1600, 3200, 6400, 12800, 25600 ng/ml) for 48 h. After the incubation duration, the viability of the cells was evaluated in accordance with the guidelines provided by the manufacturer.

### Desensitization of Caco2 and HT-29 cell lines to 5-FU

To isolate the 5-FU-resistance cancer cell subpopulation, we subjected 4 × 10^6^ Caco2 and HT-29 cells to growing dosage of 5-FU in T25 Flasks. Initiating at 25% of the IC50 value, the exposure doubled in dosage with each cycle consisting of exposure to the specific concentration of drug for four days, followed by one day of recovery in a drug-free medium, trypsinization followed by two days of recovery. This cycle lasted for a total of seven days. We repeated this process for seven cycles, resulting in a final exposure amount of 128 times the initial concentration.

### Spheroid culture of Caco2 and HT-29 cells

According to our previous study [18], HT-29 and Caco2 spheroids were generated utilizing the hanging droplet and free-floating spheroids methodology, respectively. In order to enhance the population of Cancer Stem Cells (CSCs), detachment of cells was carried out employing a solution of 0.05% trypsin/EDTA. Subsequent to inactivating trypsin, the isolated cells were subjected to two washes with Phosphate Buffered Saline (PBS) and subsequently suspended in serum-free media that had been pre-warmed for use. These cells were suspended at a concentration of either 5 or 10 thousand cells per 25µL of the serum-free medium (DMEM/F12) supplemented with 20 ng/mL EGF, 1% non-essential amino acids, 10 ng/mL bFGF, 2% B27 supplement, and 2 mM L-glutamine. Around 60 droplets of 25µL each were dispensed onto the inverted covers of 9 cm dishes. The covers were then positioned on the dishes that were filled with 5 ml of PBS in advance to maintain optimal humidity levels, following which the dishes were placed in an incubator for a duration of 96 h. Subsequently, the droplets were delicately rinsed with 2 mL of media and the resulting spheroids were translocated to poly-2-hydroxyethyl methacrylate (poly-HEMA) coated dishes for an additional incubation period of 6 days. Single-cell suspensions were added to dishes coated with poly-HEMA at different cell densities (from 1 to 5 × 10^5^ cells/mL) using the previously mentioned serum-free medium in order to grow free-floating spheroids. The cultures were then allowed to grow for a maximum of 10 days. To support the growth and viability of the spheroids, extra doses of 2% B27, bFGF, and EGF were added to the culture medium every other day.

### CFSE proliferation assay

The CFSE proliferation assay was performed using the CFSE Cell Division Tracker Kit. Parental and 5-FU exposed CRC Cells were harvested, counted, and resuspended at a concentration of 1 × 10⁶ cells/mL in the CFSE working solution. The suspension was incubated at room temperature for 20 min, protected from light. The staining reaction was quenched by adding five times the staining volume of complete medium containing 10% FBS. Labeled cells were centrifuged, washed, and resuspended in pre-warmed complete medium. To ensure recovery, cells were incubated for an additional 10 min at 37 °C. After labeling, CFSE-labeled cells were seeded and cultured under standard conditions. Following 24 h of incubation, fluorescence intensity was measured using a flow cytometer.

### Quantitative real-time PCR analysis

Real-time PCR was used to analyze the expression of a set of genes such as *KLF4*, OCT4, *SOX2*, *NANOG*, and *C-MYC* (known as stemness genes), as well as *Vimentin*, *SNAIL1*, *TWIST1*, *N-cadherin*, *E-cadherin*, and *ZEB1* (referred to as EMT genes), and *ABCB1*, *ABCC1*, and *ABCG2* (recognized as ABC transporter genes). The process involved the extraction of total RNAs from 5-FU resistant, parental, and spheroid cells utilizing TRIzol based on guideline of manufacture. Subsequently, the quantity and quality of RNA were assessed using Nanodrop, followed by cDNA synthesis from 1 µg of total RNA employing a specific cDNA synthesis kit. The subsequent step entailed the analysis of gene expression on the Rotor-Gene Q LightCycler with cycling parameters consisting of first denaturation for three minutes at 95 °C (holding stage) and 40 cycles of 10 s at 95 °C, 10 s at 60 °C, and 20 s at 72 °C. By using the 2^−ΔCT^ method to reference the target genes to glyceraldehyde-3-phosphate dehydrogenase (GAPDH), the relative expression levels of the genes were normalized. Table 1 contains the primer sequences in detail.

**Table 1:** Real-time PCR primer sequences

Genes groups	Gene name	Primer Sequence F: 5’ → 3’ *R*: 3’ → 5’
Housekeeping gene	*GAPDH*	F-CATGAGAAGTATGACAACAGCCT R- TGAACCAATGCAACCTTCTCGA
Stemness genes	*C-MYC*	F-ACACATCAGCACAACTACG R- CCTCTTACGTTCACTCCG
	*KLF4*	F-CCTCGCCTTACACATGAAGAG R- AAAGTGTGACAGAAAGGGCTAC
	*SOX2*	F-AATGGGAGGGGTGCAAAAGAGG R- GTGGTATTGGGATGGTGTGAGT
	*NANOG*	F-AGCTACAAACAGGTGAAGAC R- GGAAATGAGAAGGGTGGTAG
	*OCT4-A*	F-GTGGAGAGCAACTCCGATG R- GTCCTGATTTGCAGAGCTGT
EMT genes	*Vimentin*	F-TCTACGAGGAGGAGATGCGG R- CADAGAGCCGTCAAGTCGG
	*SNAIL1*	F-CCAGAGTTTACCTTCCAGCA R- AGCAGGTTACGAGTAGAG
	*TWIST1*	F-TTCTCGGTCTGGAGGATGGA R- TAAGTTTCTTGCCGCCCAC
	*N-cadherin*	F-GCCCAAGACAAAGAGACCC R- CAGTCAGTGAAGGAGTCAGC
	*E-cadherin*	F-CAGGAGTCATCAGTGTGGT R- GACGTGGTTACGTTATTAGGAG
	*ZEB1*	F-CTTCTCACACTCTGGGTCTTATTC R- CATTCCTCTTCTCGCTCCAACG
ABC Transporter	*ABCG2*	F-TTCCACGATATGGATTTACGG R- CCTGATGGCTTAAGTCCCTTG
	*ABCB1*	F-GTTCAGGTGGCTCTGGATAAG R- CATTACGACTGACGATGCGA
	*ABCC1*	F-CGCCTTCGCTGAGTTCCT R- GTGTGGTGTGTCGCGT

### ROS assay

To evaluate the ROS levels, cells were seeded at an initial density of approximately 200,000 cells per well. After reaching ~ 60% confluency, representing the exponential growth phase, cells were trypsinized and resuspended in ROS Buffer. The working solution of 2′,7′-dichlorofluorescein diacetate (DCFDA) reagent (10 µL of DCFDA reagent diluted in 10 mL of ROS Buffer) was prepared as per the kit instructions. Subsequently, 100 µL of working solution was added to the cells, and they were incubated for 10 min at 37 °C in the dark to ensure optimal uptake and intracellular de-esterification of the reagent (*n* = 3). After incubation, cells were washed twice with ROS Buffer to remove excess reagent and resuspended for flow cytometric analysis.

### Flow cytometry

Flow cytometry was utilized to evaluate the proportion of CSC marker expression in drug-exposed HT-29 and Caco2 cells in relation to their parental counterparts and spheroids. The cells within each cohort were detached employing trypsin/EDTA and subsequently rinsed twice with PBS. Quantification of the dissociated cells was carried out through the Trypan blue exclusion assay, with a preference for cells exhibiting viability exceeding 95% for subsequent assessment of CSC marker expression. The antibodies targeting CD44 (1:30), CD133 (1:300), and CD166 (1:90) were utilized. The incubation with these antibodies was performed with 3 × 10^5^ cells for 30 min at 4 °C. A secondary antibody, goat anti-rabbit IgG-FITC (1:100), was employed. The analysis of the percentage of cells positive for CSC markers was executed using an Attune NxT flow cytometer, with data interpretation facilitated by FlowJo VX software.

### Wound scratch assay

To assess cell migration, a wound scratch assay was conducted [25]. Cells were cultured in 35-mm dishes until they reached 90% confluence. A linear scratch was introduced at the center of the cell monolayer using a 10-µL sterile plastic pipette tip. Detached cells and debris were removed by gently rinsing the wells with PBS. After a 24-hour incubation period, the migration of parental, spheroid-derived, and 5-FU-exposed CRC cells into the scratch area was evaluated under an inverted microscope. Images were captured to record the extent of migration, and the scratch area was quantitatively analyzed using WimScratch software (Onimagin Technologies SCA, Córdoba, Spain).

### Bioinformatics and pathway analysis

To elucidate the biological mechanisms underlying the response of CRC cells to 5-FU treatment and the enrichment of CSCs, we employed various bioinformatics and pathway analysis techniques. Microarray data processing and analysis of differential gene expression were carried out using Python programming language. Differential expression analysis was conducted based on fold change criteria, where genes exhibiting a fold change greater than 2.0 (upregulated) or less than 0.5 (downregulated) were considered significant and selected for further investigation. Pathway and network analysis showed the biological processes and pathways related to the identified differentially expressed genes (DEGs). The Enrichr Python package was utilized to conduct enrichment analysis of Gene Ontology (GO) terms. DEGs were mapped to GO terms in order to determine enriched biological processes relevant to CRC and the response to 5-FU exposure. The enrichment analysis of biological pathways was conducted by leveraging the Kyoto Encyclopedia of Genes and Genomes (KEGG) database [26]. In order to find substantially enriched pathways connected to the pathophysiology of CRC and its response to 5-FU treatment, DEGs were mapped to KEGG pathways.

### Statistical analysis

The findings, which were obtained from three to four different trials, were shown as the mean value for each cohort plus or minus the standard deviation. By utilizing the analysis of variance (ANOVA) method along with Tukey’s post hoc analysis, comparisons between the three experimental sets were made. For the Windows operating system, GraphPad Prism version 8.0 was used to perform the statistical analyses. To signify statistical significance, a threshold of less than 0.05 was set.

## Results

### The sensitivity of Caco2 and HT-29 cell lines to 5-FU

**Fig. 1:**
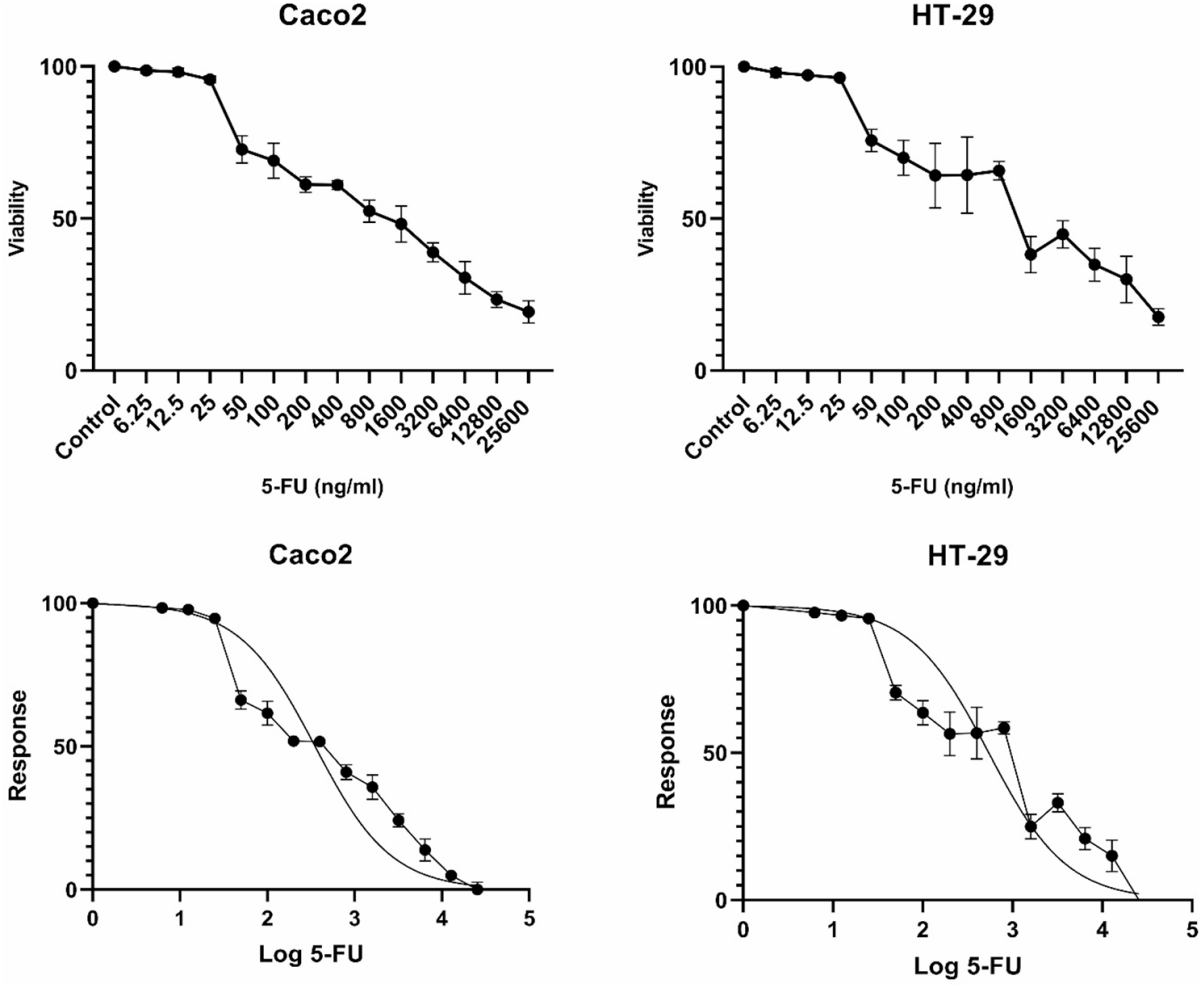
Response of Caco2 and HT-29 CRC cell lines to cytotoxic effects of 5-FU. Caco2 and HT-29 cells exhibited cytotoxic responses at concentrations of 50 ng/ml or higher of 5-FU. The IC50 values for 5-FU in Caco2 and HT-29 cells were determined to be 353.4 ng/ml and 543.3 ng/ml, respectively

In order to evaluate the responsiveness of Caco2 and HT-29 cancer cells towards 5-FU, a study was carried out involving the utilization of an MTT assay to determine cell survival rates subsequent to varying levels of exposure to 5-FU (ranging from 6.25 to 25,600 ng/ml). Our results indicated that both Caco2 and HT-29 cells exhibited sensitivity to 5-FU. Specifically, Caco2 and HT-29 cells displayed cytotoxic effects at concentrations equal to or greater than 50 ng/ml of 5-FU. The IC50 of 5-FU for Caco2 and HT-29 was calculated 353.4 ng/ml and 543.3 ng/ml, respectively (Fig. 1).

### Desensitization of Caco2 and HT-29 cell lines to 5-FU

**Fig. 2:**
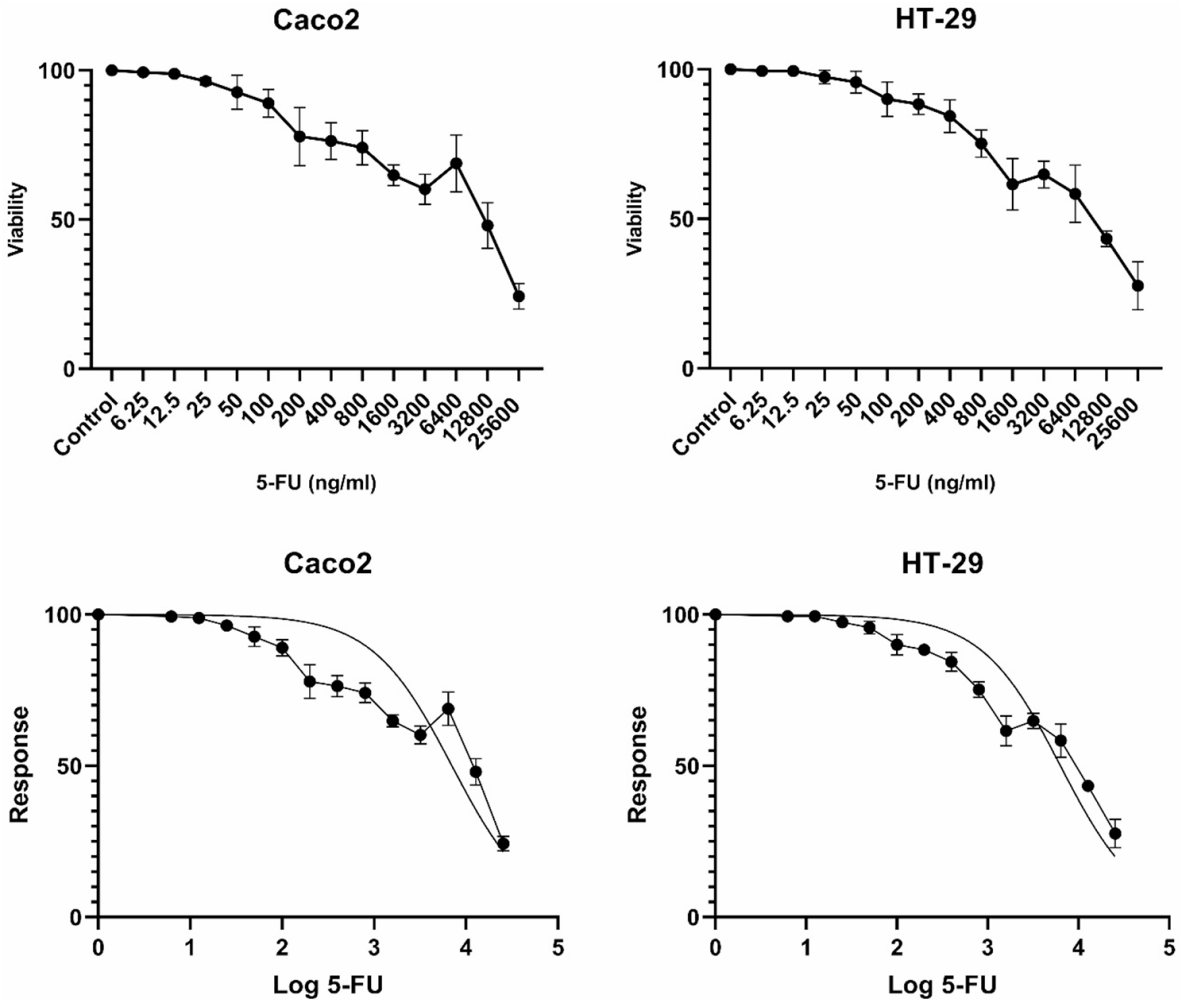
Response of 5-FU-exposed Caco2 and HT-29 CRC cells to cytotoxic effects of 5-FU. The IC50 values for 5-FU in Caco2 and HT-29 cells were determined to be 7,039 ng/ml and 6348 ng/ml, respectively

In order to isolate the cancer cell subpopulation, we subjected 4 × 10^6^ HT-29 and Caco2 cells to growing concentrations of 5-FU. Initially, the concentrations stood at 88.35 ng/ml for Caco2 and 135.8 ng/ml for HT-29, with each successive cycle doubling the dosage. This procedure was repeated for seven cycles, culminating in a final exposure concentration of 11,308.8 ng/ml for Caco2 and 17,382.4 ng/ml for HT-29. The IC50 of the isolated subpopulation was determined to be 7,039 ng/ml for Caco2 and 6,348 ng/ml for HT-29 (Fig. 2). These values represented significant increases of 19.9 and 11.68 times the IC50 of parental populations of Caco2 and HT-29 cells, respectively compared to parental cells (P-value < 0.0001).

### Morphological characteristics of spheroids and 5-FU-exposed CRC cells

We recorded the behavior of Caco2 and HT-29 cells during treatment with 5-FU considering the alterations in each cycle. At initial cycles, cell viability was decreased, leading to severe mortality and leaving only a few clusters of surviving cells. Despite this initial decline, the number of cells stabilizes over the following cycles, suggesting an equilibrium between cellular proliferation and cell death. Subsequent passages exhibit a gradual rise in the growth rate during the successive passages. This implies that the cells undergo adaptation and selection, progressively adjusting to the culture conditions.

**Fig. 3:**
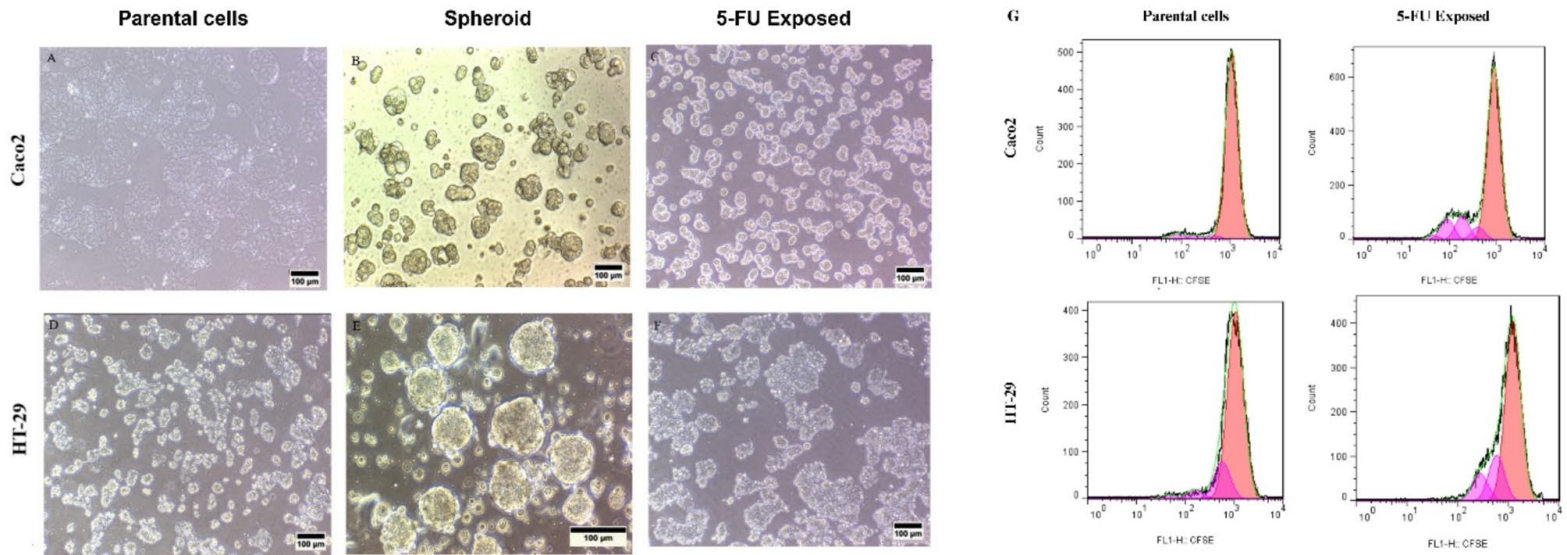
Characteristics changes in Caco2 and HT-29 spheroids and 5-FU exposed cells. (**A**) Parental Caco2 cells showed epithelial morphology, forming tightly packed monolayers resembling epithelial cells. (**B**) Spheroids derived from Caco2 cells exhibited a compact, spherical morphology. (**C**) Caco2 cells appear heterogeneous, with most cells showing cuboidal or round shapes and occasional giant cells. However, cultivation conditions favor specific cell subpopulations, altering cell morphology compared to the parental line. Drug-adapted cells display a looser structure compared to the spherical colony-shaped aggregations of parental cells. (**D**) Parental HT-29 cells display a range of shapes, including polygonal, spindle-shaped, and elongated morphologies. (**E**) Spheroids derived from HT-29 cells displayed a rounded shape and exhibited a dense, compact morphology. (**F**) There were no substantial changes noted in their morphology after exposure to 5-FU; however, they displayed a less firm attachment to the culture flask and seemed to accumulate in colony formations. (**G**) CFSE analysis showed slight increase in proliferation of both CRC cell lines after prolong treatment with 5-FU

After cell culture in standard condition, the original Caco2 cells exhibited an epithelial morphology, forming closely packed monolayers reminiscent of typical epithelial cells. In contrast, the parental HT-29 cells demonstrated a variety of shapes, encompassing polygonal, spindle-shaped, and elongated morphologies (Fig. 3A, D). Both cell lines produced 3D spheroids in serum-free media. HT-29 utilized the hanging drop method, while Caco2 employed a free-floating culture under non-adherent conditions. Microscope images of the spheroids showed that HT-29 cells produced spheroids characterized by a smooth, rounded surface and a dense, compact morphology, while Caco2 cells exhibited spontaneous formation of spheroids with a rounded structure (Fig. 3B, E). Besides, the isolated population displayed morphological and behavioral alteration (Fig. 3C, F). Both 5-FU-exposed populations showed a slight increased proliferation rate. CFSE analysis showed a division index of 0.033 and 0.179 for parental and 5-FU exposed caco2 CRCs, respectively. These amounts were 0.112 and 0.179 for parental and 5-FU exposed HT-29 CRCs, respectively (Fig. 3G).

Although isolated Caco2 cells were heterogeneous, most cells were cuboidal or round with some giant cells apparent. The cultivation conditions, however, seem to select the growth of subpopulations of cells leading to a cell morphology that differs from the parental cell line. The drug-adapted cells are rather looser compared to parental cells which grow as spherical colony-shape aggregations. Regarding the HT-29, no significant change in morphology was observed; however, they showed looser attachment to the culture flask and appeared as colony accumulation.

### Enrichment of cancer stem cell-like populations

**Fig. 4:**
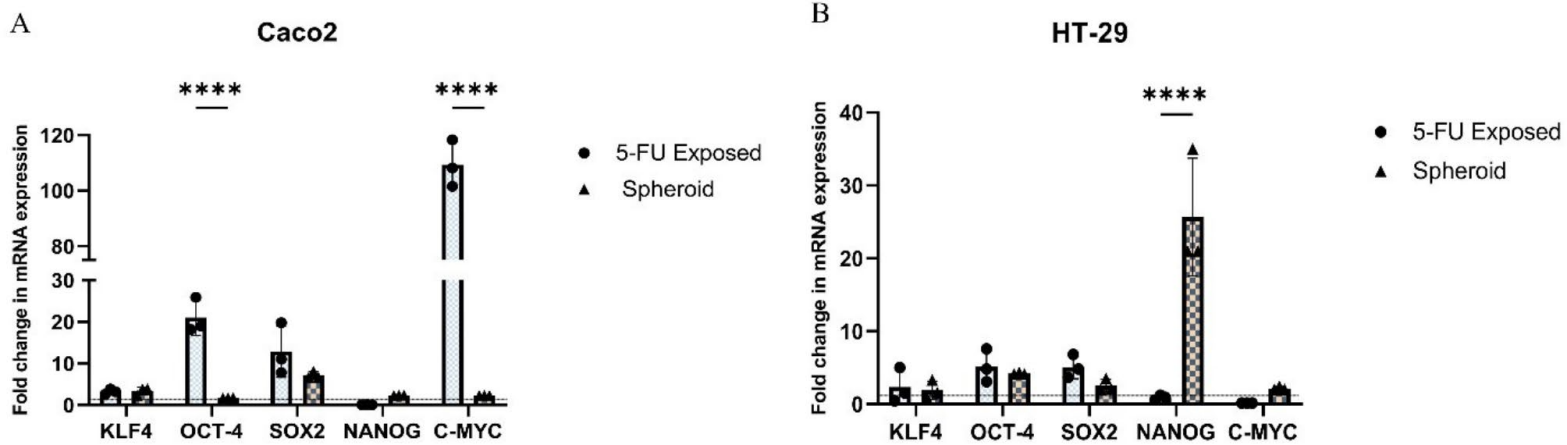
RT-qPCR analysis of stemness genes. When HT-29 and Caco2 cells were exposed to 5-FU, the expression of genes that regulate stemness was found to be higher than in the parental cell population. **A**) Compared to the spheroids, the isolated Caco2 subpopulation exposed to 5-FU showed a significant increase in *C-MYC* and *OCT4* expression (p-value < 0.0001). **B**) With the exception of *NANOG*, the expression levels of stemness genes in the isolated HT-29 subpopulation cells exposed to 5-FU did not differ significantly from those in the spheroids. Interestingly, spheroid cells showed a more marked upregulation of *NANOG* (p-value < 0.0001) in comparison to the isolated HT-29 subpopulation cells exposed to 5-FU. The baseline gene expression in parental cells is represented by a dotted line. Data are presented as mean ± SD from three independent experiments as **** = p-value < 0.0001

Crucial stemness genes such as *KLF4*,* OCT-4*,* SOX2*,* NANOG*, and *C-MYC*, which are important regulators of pluripotency and the capacity for self-renewal in CSCs, were expressed in cancer cells exposed to 5-FU in comparison to both spheroids and their parental population. When HT-29 and Caco2 cells exposed to 5-FU were compared to the parental population, real-time PCR analysis confirmed a significant upregulation of the examined stem cell-associated genes, with the exception of *NANOG* in Caco2 and *C-MYC* and *NANOG* in HT-29. Furthermore, the 5-FU-exposed isolated Caco2 subpopulation exhibited a notable upregulation of *C-MYC* and *OCT4* compared to the 3D spheroids (p-value < 0.0001) (Fig. 4A). However, the expression of stemness genes in the 5-FU-exposed isolated HT-29 subpopulation cells did not significantly differ from that of the spheroids, except for *NANOG*, where spheroid cells demonstrated a more pronounced upregulation compared to the 5-FU-exposed isolated HT-29 subpopulation cells (p-value < 0.0001) (Fig. 4B).

**Fig. 5:**
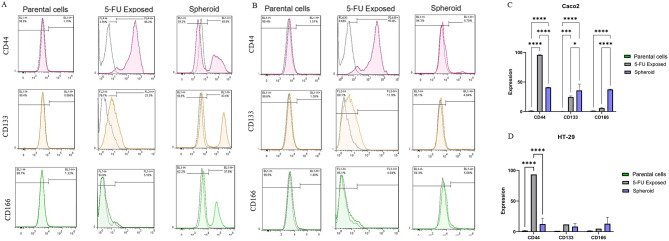
Flow cytometry analysis of CRC stemness markers expression. The expression of CRC-CSC markers in HT-29 and Caco2 subpopulations exposed to 5-FU was analyzed using flow cytometry in comparison to their parental and spheroid cells. The changes in marker expression were confirmed by flow cytometry, specifically in CD44 and CD133 in Caco2 subpopulations exposed to 5-FU (**A**, **C**) and in CD44 in HT-29 subpopulations exposed to 5-FU (**B**, **D**). Data are presented as mean ± SD as *** = p-value < 0.001 and **** = p-value < 0.0001

To strengthen these findings, we examined the expression of CSC surface markers CD166, CD44, and CD133 using flow cytometry. Consistent with our observations from the analysis of stemness gene expression, surface marker analysis also demonstrated the alteration of CD166, CD44, and CD133 CSC markers. In Caco2 cells exposed to 5-FU, CD44 was upregulated compared to parental and spheroid cells; however, CD133 was upregulated compared to parental cells and CD166 expression did not show any change (Fig. 5A, C). Besides, HT-29 cells exposed to 5-FU only showed an upregulation in CD44 surface markers (Fig. 5B, D). Table 2 shows the expression percentage of CRC stemness surface markers, CD44, CD166, and CD133, in 5-FU exposed compared to their parental cells and spheroid with all significant differences and P-values.

**Table 2:** The percentage of CRC stemness surface markers, CD44, CD166, and CD133, expressed in cells exposed to 5-FU and spheroids, in comparison to their parental cells

Marker	CRC cell line					
	Caco2			HT-29		
	Parental	5-FU Exposed	Spheroid	Parental	5-FU Exposed	Spheroid
CD44	1.325 ± 0.24	95.99 ± 0.29^****^	40.92 ± 0.17^****^	1.685 ± 0.16	94.19 ± 1.11^****^	12.5 ± 9.61
CD133	0.53 ± 0.07	24.6 ± 1.83^***^	35.82 ± 10.71^****^	1.365 ± 0.02	12.85 ± 1.34	8.345 ± 4.81
CD166	1.285 ± 0.04	5.8 ± 0.98	37.47 ± 0.45^****^	1.57 ± 0.12	5.42 ± 0.67	13.05 ± 10.45

The data are presented as the mean ± SD. Significant upregulation of CSC markers indicated as *** = p-value < 0.001, **** = p-value < 0.0001

### Enriched 5-FU-resistant CRC cells showed potent scavenging systems

To investigate the redox balance and scavenging capacity of enriched 5-FU-resistant CRC cells, mediated by several factors including ALDH—a marker and functional contributor to maintaining CSC properties—we measured ROS levels using flow cytometry. Parental Caco2 and HT-29 cells, along with their spheroid-derived and 5-FU-exposed counterparts, were assessed to evaluate the role of ROS scavenging systems in resistance mechanisms.

**Fig. 6:**
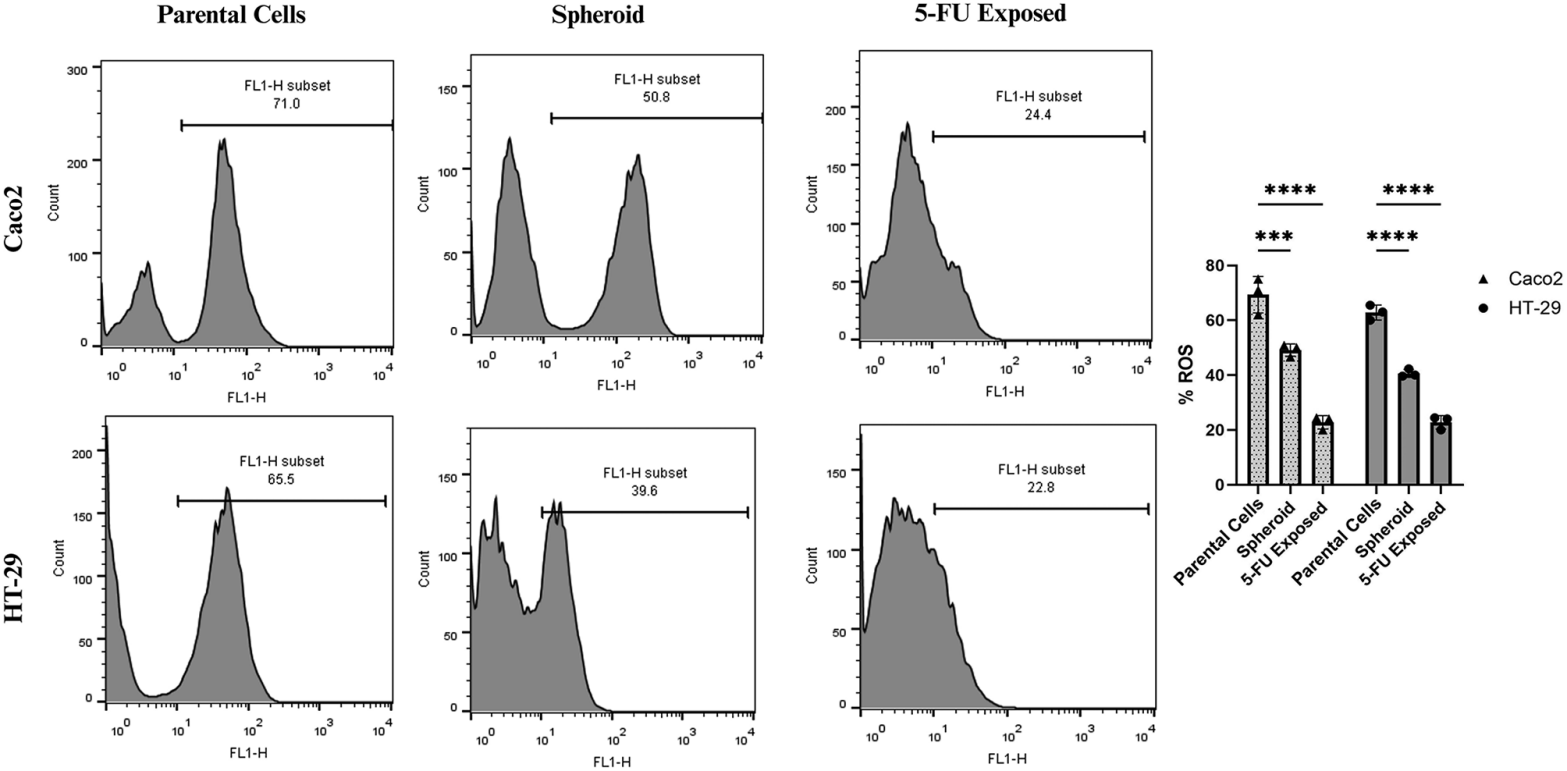
Analysis of ROS levels. The parental cells exhibit the highest ROS levels, followed by spheroid cells, with the lowest levels observed in 5-FU exposed cells. Data are presented as mean ± standard deviation (SD) from three independent experiments. Statistical significance is indicated as ***p < 0.001, ****p < 0.0001

The results revealed a significant reduction in ROS levels in spheroid-derived and 5-FU-exposed cells compared to the parental cells. In Caco2 cells, the percentage of ROS-positive cells was 71.0% in parental cells, decreasing to 50.8% in spheroid cells (*p* < 0.001) and 24.4% in 5-FU-exposed cells (*p* < 0.0001). Similarly, in HT-29 cells, ROS-positive populations decreased from 65.5% in parental cells to 39.6% in spheroid cells (*p* < 0.0001) and 22.8% in 5-FU-exposed cells (*p* < 0.0001) (Fig. 6). These findings indicate that both spheroid CSCs and 5-FU-resistant CRC cells exhibit enhanced ROS scavenging capacity, potentially enabling their survival under oxidative stress.

**Table 3:** The IPA pathways linked to the shared differentially expressed genes in 5-FU exposed subpopulations of CRC cells

Gene set	Pathway	Overlap	*P*-value	Adjusted *P*-value	Odds Ratio	Combined Score	Genes
KEGG_2019_Human	MicroRNAs in cancer	3/299	0.000847	0.011005	19.95709	141.1835	*ABCB1; ZEB1; CD44*
KEGG_2019_Human	Pluripotency of stem cells	2/139	0.003557	0.023123	26.34373	148.545	*SOX2; KLF4*
KEGG_2019_Human	Proteoglycans in cancer	2/201	0.007288	0.031581	18.07949	88.97873	*TWIST1; CD44*

### 5-FU-resistant CRC cells were driven toward the EMT

**Fig. 7:**
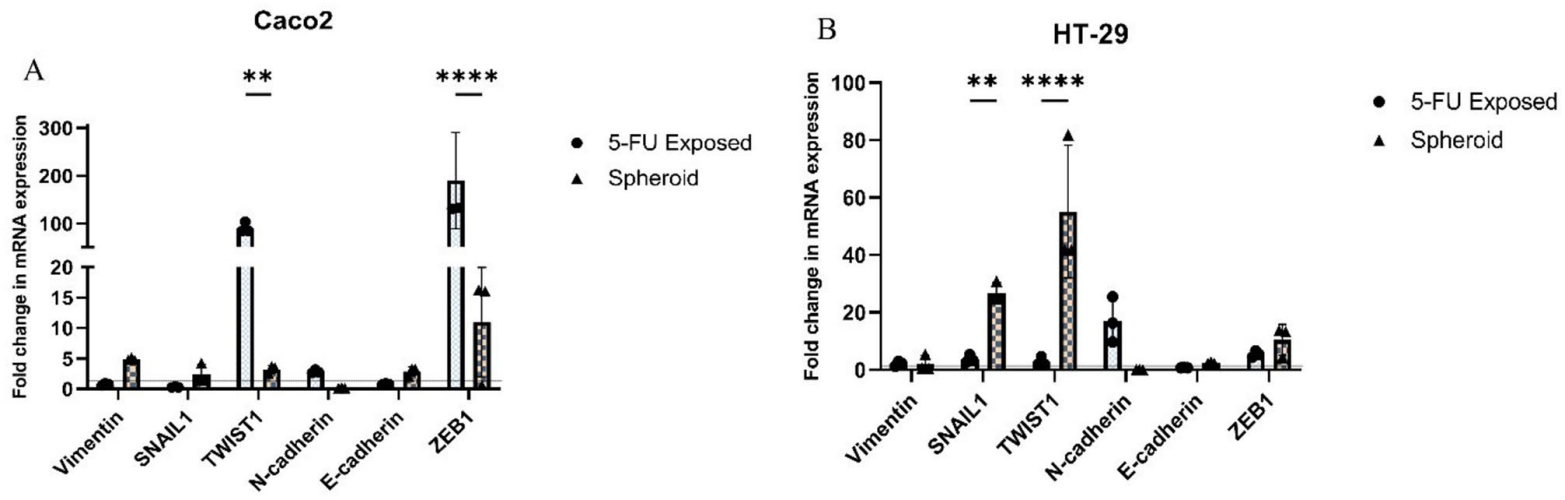
RT-qPCR analysis of EMT genes. In both 5-FU-treated Caco2 and HT-29 cells, there was a notable shift in the expression of genes related to EMT. In Caco2 cells, *TWIST1*,* ZEB1*, and *N-cadherin* were significantly increased, while *SNAIL1*,* Vimentin*, and *E-cadherin* were decreased following exposure to 5-FU. Importantly, *TWIST1* (p-value = 0.0073) and *ZEB1* (p-value < 0.0001) were markedly upregulated in 5-FU-exposed Caco2 cells compared to spheroids. Similarly, in HT-29 cells, all EMT-inducing genes were upregulated, while *E-cadherin* was downregulated. Notably, *TWIST1* (p-value < 0.0001) and *SNAIL1* (p-value = 0.0049) were significantly elevated in spheroids compared to 5-FU-exposed HT-29 cells. The baseline gene expression in parental cells is represented by a dotted line. Data are presented as mean ± SD from three independent experiments as **** = p-value < 0.0001, ** = p-value < 0.01

To delineate the EMT properties within distinct subpopulations, we conducted a comparative analysis of the expression patterns of EMT-promoting genes, namely *TWIST1*,* SNAIL1*,* ZEB1*,* Vimentin*,* E-cadherin*, and *N-cadherin*, in both 5-FU-exposed isolated CRC cells and spheroids. In 5-FU-exposed isolated Caco2 cells, the upregulation of genes that facilitate EMT such as *TWIST1*,* ZEB1*, and *N-cadherin* was elevated compared to parental cells, while *SNAIL1*,* Vimentin*, and *E-cadherin* exhibited downregulation. Notably, *TWIST1* (p-value = 0.0073) and *ZEB1* (p-value < 0.0001) were significantly upregulated in 5-FU-exposed isolated Caco2 cells compared to spheroids (Fig. 7A). Similarly, in 5-FU-exposed isolated HT-29 cells, all EMT-related genes, including *TWIST1*,* SNAIL1*,* ZEB1*,* Vimentin*, and *N-cadherin*, showed upregulation than parental cells, while the tumor and EMT suppressor protein *E-cadherin* experienced downregulation. Particularly noteworthy was the significant upregulation of *TWIST1* (p-value < 0.0001) and *SNAIL1* (p-value = 0.0049) in spheroids compared to 5-FU-exposed isolated HT-29 cells (Fig. 7B).

**Fig. 8:**
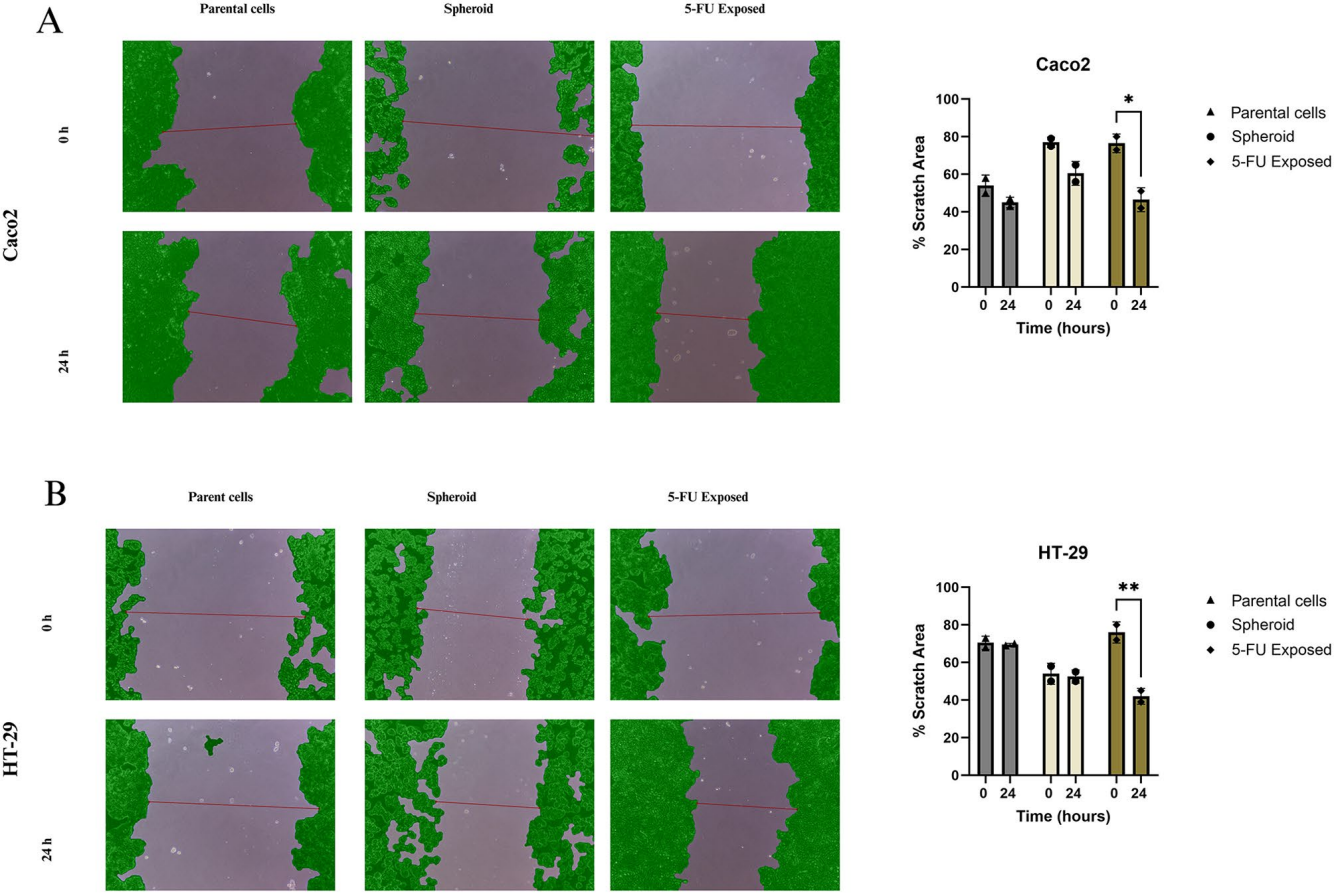
Wound scratch assay for migration assessment. Images of wound areas for Caco2 (**A**) and HT-29 (**B**) cells at 0 h and 24 h post-scratch are shown. The cell areas are highlighted in green, with the mean scratch length marked by a red line. The bar graphs on the right represent the percentage of the remaining scratch area at 0 and 24 h. For both Caco2 and HT-29 cells, 5-FU exposed CRC cells exhibited significant wound closure, indicating higher migratory capacity. However, migration capacity of parental and spheroid cells was insignificant. Data represent mean ± SD from two independent experiments. Statistical significance: *p < 0.05 and **p < 0.01

The wound scratch assay was performed to evaluate the migration ability of parental, spheroid-derived, and 5-FU-exposed CRC cells. The 5-FU-exposed caco2 cells demonstrated considerable migration into the wound area, with a significant reduction in the percentage of the wound area after 24 h (*p* < 0.05). However, parental and spheroid-derived caco2 cells exhibited insignificant migration during 24 h (*p* = 0.86, *p* = 0.2, respectively) (Fig. 8A). Similarly, in HT-29 cells, the 5-FU exposed group displayed the highest migratory ability, with notable migration after 24 h (*p* < 0.01). The spheroid-derived and parental cells exhibited insignificant migration (*p* = 0.99). These results suggest that 5-FU-resistant CRC exhibits higher migration ability associated with chemoresistance and cancer stem cell-like properties (Fig. 8B).

### 5-FU-resistant CRC cells exhibited altered expression of multi-drug resistant (MDR) genes

**Fig. 9:**
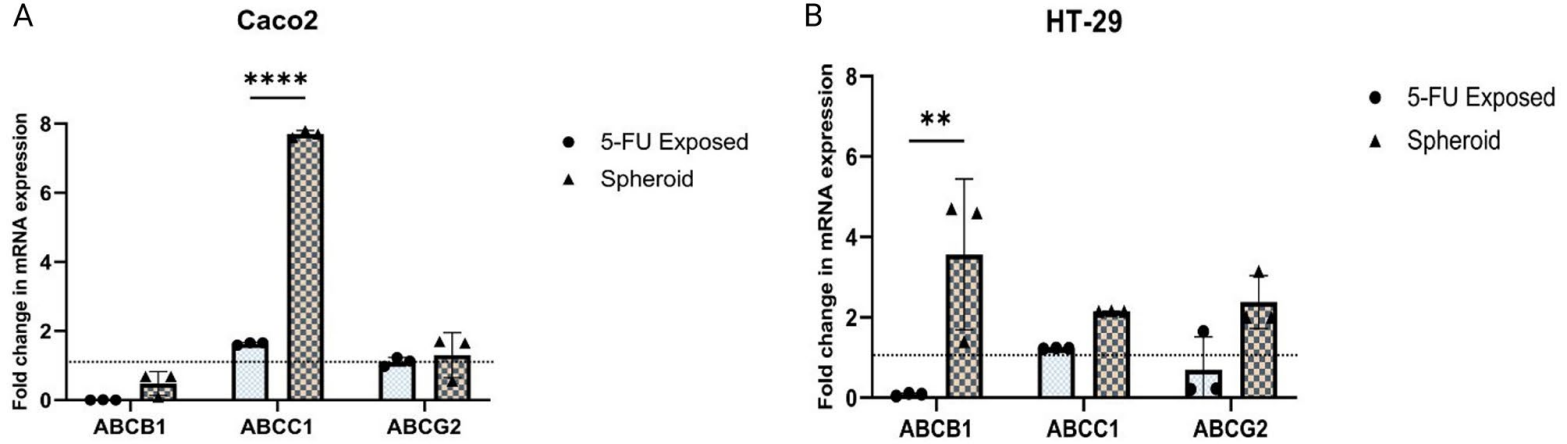
RT-qPCR analysis of MDR genes. **A**) Compared to parental cells, 5-FU-treated Caco2 cells showed a moderate uptick in *ABCB1* and *ABCC1* expression. Interestingly, spheroids showed a significantly stronger upregulation of *ABCC1* than did isolated 5-FU-exposed Caco2 cells (p-value < 0.0001). **B**) In contrast, HT-29 cells exposed to 5-FU showed only a modest increase in *ABCC1* expression when compared to the parent HT-29 cells. HT-29 cells exposed to 5-FU showed significantly lower *ABCB1* expression than spheroids (p-value = 0.0012). The baseline gene expression in parental cells is represented by a dotted line. Data are presented as mean ± SD from three independent experiments as ****= p-value < 0.0001, ** = p-value < 0.01

Another essential feature of CSCs is MDR, which is frequently enabled by a collection of ABC proteins. In order to compare the expression profiles of these important MDR genes—ABC1, *ABCB1*, and *ABCG2*—in CRC cells exposed to 5-FU with those of parental cells and 3D spheroids, we looked into these. Specifically, 5-FU-exposed cells derived from Caco2 showed a modest increase in *ABCB1* and *ABCC1* expression than parental cells. Notably, the upregulation of *ABCC1* was significantly more pronounced in spheroids than in 5-FU-exposed Caco2 cells (p-value < 0.0001) (Fig. 9A). In contrast, HT-29-derived 5-FU-exposed cells exhibited a slight elevation in *ABCC1* expression only compared to HT-29 parental cells. However, the expression of *ABCB1* was notably more prominent in spheroids compared to isolated 5-FU-exposed HT-29 cells (p-value = 0.0012) (Fig. 9B).

### Gene ontology (GO) analysis

#### Enriched molecular functions in CRC cell lines in response to 5-FU treatment

In our analysis of differentially expressed genes, we identified several molecular functions that were significantly enriched based on GO terms. The most prominently enriched molecular functions included E-box binding (GO:0070888), transcription regulatory region nucleic acid binding (GO:0001067), DNA-binding transcription factor binding (GO:0140297), glycerophospholipid flippase activity (GO:0140333), ABC-type xenobiotic transporter activity (GO:0008559), hyaluronic acid binding (GO:0005540), RNA polymerase II cis-regulatory region sequence-specific DNA binding (GO:0000978), transcription cis-regulatory region binding (GO:0000976), phosphatidylethanolamine flippase activity (GO:0090555), and phosphatidylcholine floppase activity (GO:0090554). These molecular functions were identified as significantly enriched based on their respective -log10 (Adjusted P-value) scores. This comprehensive analysis help understanding the molecular mechanisms driving CRC development and the cellular response to 5-FU treatment.

#### Enriched biological pathways in CRCs in response to 5-FU treatment

**Fig. 10:**
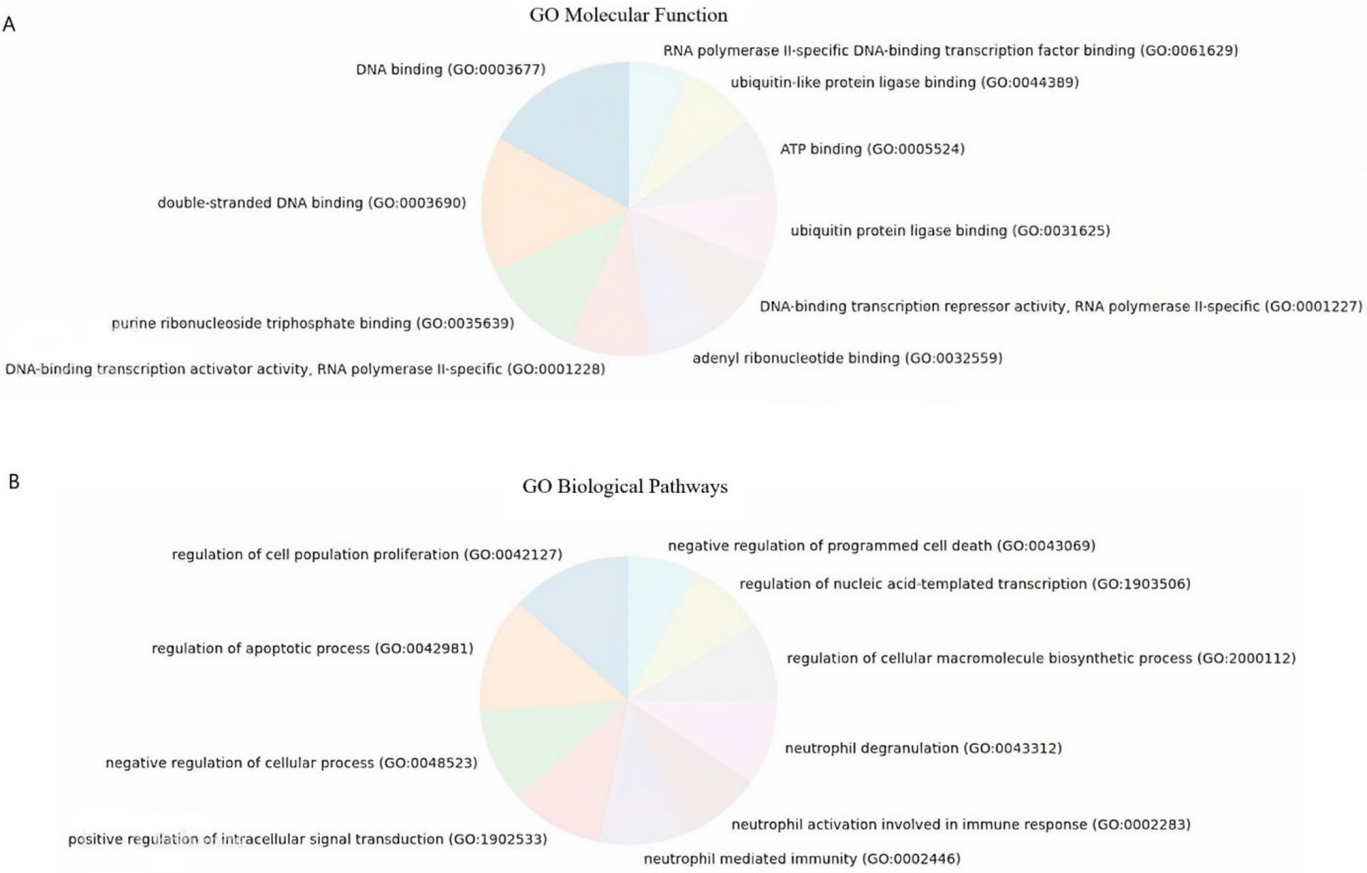
The most important enriched gene ontologies are based on (**A**) molecular functions and (**B**) biological pathways

According to the KEGG database analysis, the common enriched biological pathways observed in both Caco2 and HT-29 cells from parental and 5-FU-exposed subpopulations include the control of cysteine-type endopeptidase activity implicated in the apoptotic process, inhibition of the DNA damage response, transmission of signals by the p53 class mediator, and suppression of signal transduction by the p53 class mediator. (Fig. 10).

#### Overlap of differentially expressed genes in CRC cell lines in response to 5-FU treatment

**Fig. 11:**
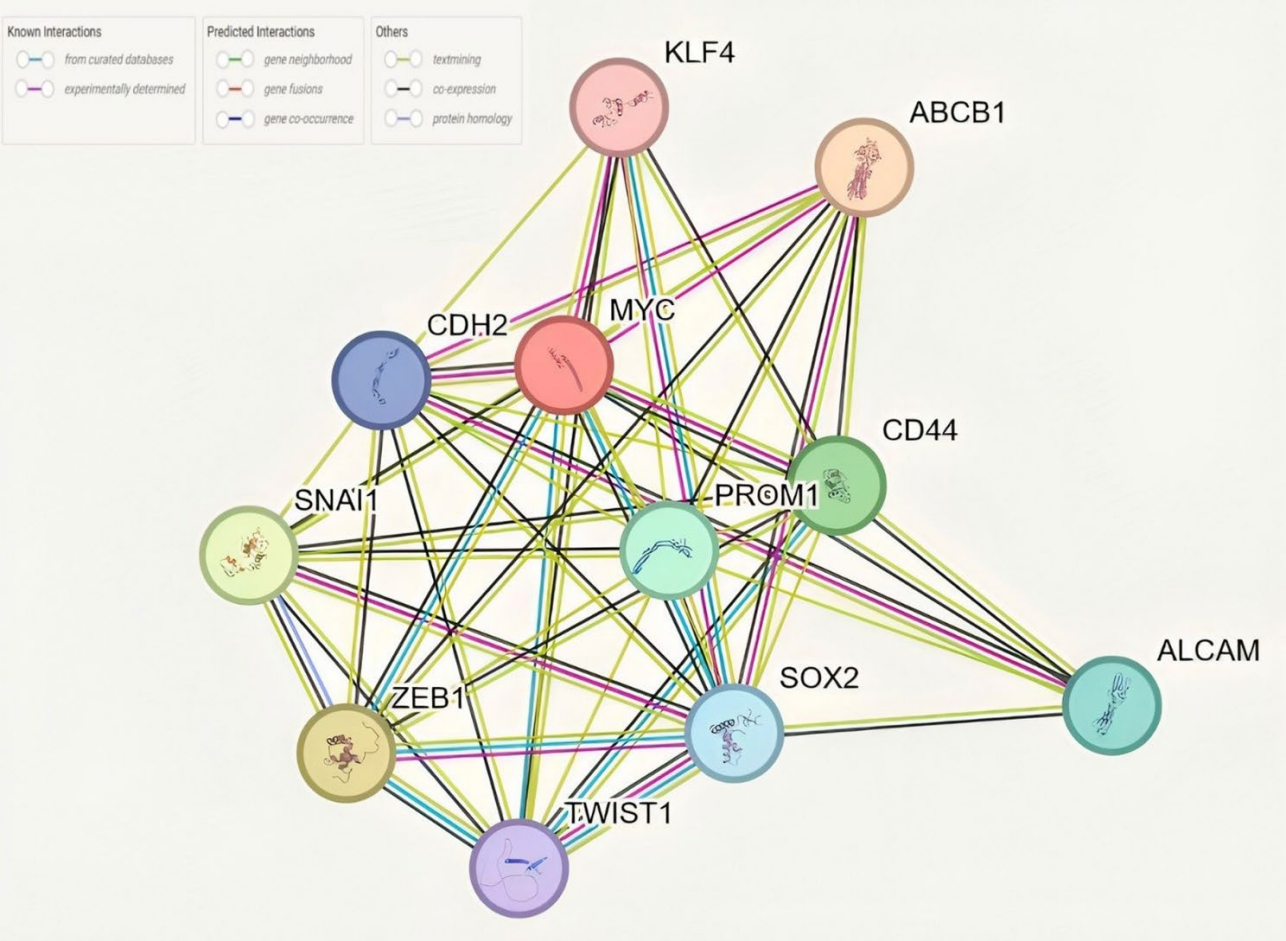
Gene interaction network of common differentially expressed genes. The network illustrates the interactions between 11 genes significantly enriched in KEGG pathways, including MicroRNAs in cancer, signaling pathways regulating pluripotency of stem cells, and Proteoglycans in cancer. Each node represents a gene, and the edges represent known or predicted interactions, categorized as experimentally determined (pink), curated from databases (blue), or predicted through gene neighborhood, co-expression, and text mining (green, black, and yellow respectively). Key players such as MYC, CD44, PROM1, and SOX2 are central to the network, indicating their collaborative roles in the pathways analyzed

Analysis of common differentially expressed genes in both parental and 5-FU-exposed subpopulations of Caco2 and HT-29 cells revealed a highly significant overlap with enriched pathways in the KEGG database, including MicroRNAs in cancer, signaling pathways regulating pluripotency of stem cells, and Proteoglycans in cancer (Table 3). Also, a network consisting of 11 genes was found to collaborate in these pathways (Fig. 11). This association was indicated by -log10 (Adjusted p-value) using a significance threshold of p-value less than 0.05.

## Discussion

In order to address the challenge of treatment failure caused by cancer progression, recurrence, and metastasis, which are linked to the biological characteristics of CSCs and their disruption of inherent drug resistance mechanisms, creating suitable pre-clinical models emerges as a critical priority for testing targeted therapies [27]. Considering the resistant nature of CSCs to conventional cancer treatments, using chemotherapeutic drug exposure protocol may arise as a new avenue for isolating subpopulations displaying stem-like features. Previous studies indicated that various methods for isolating CSCs result in distinct morphological and functional traits in isolated subpopulations depending on the approach employed [18, 28, 29]. Establishing CSC isolation techniques grounded in the clinical principles of CSC evolution, such as natural selection during chemoradiotherapy, would furnish a suitable in vitro model for assessing potential diagnostic and therapeutic strategies targeting CSCs. Some studies have highlighted the natural isolation of CSCs after radiochemotherapy in cancer patients causing tumor recurrence, sometimes many years later [30, 31]. It has been shown that short-term methotrexate treatment enriches CSCs by eliminating sensitive cells and promoting the survival of a resistant subset [32]. Therefore, simulating such a clinical condition in vitro with chemotherapeutic agents would provide more efficient CSC models to be applied for preclinical experiments. In this regard, evaluating the morphological characteristics, CSC attributes, and patterns of gene expression associated with pluripotency, EMT, and drug resistance is crucial for selecting the most suitable approach.

In this study, a 5-FU exposure method in 2D cell culture was employed and juxtaposed against the 3D spheroid formation approach to enrich CSCs from Caco2 and HT-29 CRC cell lines. We employed 5-FU as the standard chemotherapeutic regimen for CRC as the treatment drug [33]. Besides, several studies have documented that CRC cell subpopulations exhibiting stem-like traits tend to develop resistance to 5-FU [34–36]. The isolation protocol involved treatment-recovery intervals designed to support the drug-exposed cells in proliferating and sustaining their population. Upon reaching the steady proliferation stage (after the seventh cycle), we evaluated the IC_50_ of isolated subpopulations that showed an increase in IC_50_ for both 5-FU-exposed Caco2 (19.9 times of parental population) and HT-29 (11.68 times of parental population) cell lines. The results showed that isolated subpopulations indicating more resistance features compared to parental cells. Considering the enrichment approach, the resistant feature of established CSC model can be utilized in experiments evaluating the effects of anticancer agents on CSCs in in vitro and preclinical studies.

Since gene expression, as well as surface markers, are the main features to characterize CSCs, we evaluated specific CSC-related gene expression and surface markers as well as ROS levels. Firstly, we carried on stemness gene expression analysis by real-time PCR on *KLF4*,* OCT4*,* SOX2*,* NANOG*, and *C-MYC* genes. Our observations showed that 5-FU-exposed Caco2 (*KLF4*,* OCT4*,* SOX2*, and *C-MYC*) and HT-29 (*KLF4*,* OCT4*, and *SOX2*) cells upregulated stemness genes. Besides, *OCT-4* and *C-MYC* upregulation was significantly higher in the 2D culture of 5-FU-exposed Caco2 cells than in the 3D spheroid population. *OCT-4* and *C-MYC* function as transcription factors that maintain the pluripotency and stemness of CSCs. Besides, OCT-4 activation represents a mechanism for activating the *C-MYC* oncogene [37]. The notable expression of stemness genes, particularly *OCT-4* and *C-MYC*, within our 5-FU-exposed CRC cells, may serve as an indicative marker for their self-renewal potential. It has been shown that carboplatin induces breast CSC enrichment via hypoxia-inducible factor (HIF)-dependent pathways, increasing pluripotency markers such as *KLF4* and *SOX2* and activating survival signaling mechanisms [38]. In addition, increased expression of stemness markers *OCT4* and *SOX2* in Doxorubicin-resistant triple negative breast cancer cells has been observed, supporting the role of these genes in maintaining CSC traits across cancer types [39]. The increased expression of *OCT4* in osteosarcoma sarcospheres directly supports our observations of upregulated stemness genes in 5-FU-resistant CR CSCs, reinforcing the conserved role of these genes across different cancers and different mechanism of enrichment [40]. So, these results suggest that 5-FU exposure induces selective pressure, allowing only resistant CSC-like cells to survive and expand.

Studies have indicated that the levels of ROS in CSCs play a crucial dual role. Low ROS levels have been identified as a key marker of CSCs [41, 42]. This low ROS state, facilitated by enhanced antioxidant systems such as glutathione synthesis and metabolic reprogramming, supports CSC survival, stemness, and resistance to therapy [41, 43]. Additionally, ALDH, a robust CSC biomarker, correlates with ROS regulation in CSCs [44]. High ALDH activity reduces ROS levels by enhancing antioxidant pathways, including NRF2-mediated expression of enzymes like GPX3 and SOD-2, further promoting CSC chemoresistance and therapeutic evasion [45, 46]. This dual role of ROS and ALDH highlights their importance as potential biomarkers and therapeutic targets in CSCs. Thus, we assessed ROS levels as potential biomarker for CSCs and also a as a proxy for evaluating ALDH activity. Our analysis revealed a significant reduction in ROS levels in 5-FU-resistant cell populations, consistent with the previous studies linking low ROS to CSC properties. A study by Tsochantaridis et al. showed that overexpression of *ALDH1B1* altered cell morphology and enhanced resistance to chemotherapeutics like doxorubicin and 5-FU. It also promoted migration and EMT through *ZEB1/vimentin* upregulation and *E-cadherin* downregulation [46]. These observations align with ours regarding the enrichment of 5-FU-resistant CRC cells, showing similarities in morphological alterations, enhanced EMT, increased migration, and elevated *ZEB1* expression which supports that ROS reduction can also reflect the higher activity of scavenging system including ALDH, as a viable biomarker for CSCs. Collectively, our results support the hypothesis that CSCs rely on metabolic adaptations to resist oxidative stress, a trait linked to therapy resistance.

In addition, we evaluated the surface CSC markers, including CD44, CD133, and CD166, on the 5-FU exposed populations. The findings indicated a noteworthy augmentation in CD44 and CD 133 in 5-FU-exposed Caco2. Besides, HT-29 cells showed a significant increase in CD44-positive cells. CD44 acts as a transmembrane adhesion receptor, binding to hyaluronic acid in the extracellular matrix (ECM), thus playing a pivotal role in matrix adhesion and cellular aggregation within the cellular microenvironment. Additionally, CD133 and CD44 enhance clonal formation capacity in CRC and are associated with CSC properties [47, 48]. Several studies have emphasized the critical role of CD44 and CD133 in CRC progression and CSC features. A strong correlation between the mRNA expression of CD44 and CD133 and CRC metastasis has been demonstrated [48, 49]. Therefore, the results of surface marker expression on 2D 5-FU-exposed CRC showed that this isolation approach has the potential to enrich CSC populations. Given the increasing focus on discovering novel and efficient therapeutic targets within CSCs, employing our CSC model would demonstrate the effectiveness of targeting drugs for resistant and invasive tumor populations, owing to the model’s inherent invasiveness and resistance traits [50].

To look into the connection between the expression levels of EMT-related genes and the enhanced CSC characteristics within 5-FU-exposed CRC cells, we compared the expression of *SNAIL1*,* TWIST1*,* Vimentin*,* ZEB1*,* E-cadherin*, and *N-cadherin*. The results showed a significant increase in pro-EMT genes such as *TWIST1*,* ZEB1*, and *N-cadherin* in 5-FU-exposed Caco2 compared to parental populations. It is notable that *TWIST1* and *ZEB1* significantly upregulated in 5-FU-exposed Caco2 compared to the spheroid population. Besides, HT-29 cells showed a significant increase in all EMT-related genes. Notably, *E-cadherin* was downregulated in both 5-FU-exposed Caco2 and HT-29 populations. It has been demonstrated that during the process of EMT in cancers, *N-cadherin* undergoes upregulation, whereas *E-cadherin* experiences downregulation. This transition, known as the “cadherin switch,” correlates with heightened migratory and invasive characteristics, ultimately leading to lower patient survival rates [51]. So, the isolated population by 5-FU exposure was much more invasive than their parental cells. We also envaulted the phenotypic features of cadherin switch by performing migration assay. Our results showed that unlike parental and spheroid CRC cells, 5-FU exposed CRC cells gained more potent migratory ability during enrichment process which correlate with genetic alteration observed in *E-cadherin* and *N-cadherin* levels. These results showed that using 5-FU exposure methods enhances EMT features in isolated CSCs compared to spheroid formation. The EMT stands out as a critical hallmark of CSCs, driving metastasis and invasion. Our findings demonstrated that enriching CSCs through exposure to chemotherapeutic agents resulted in a model exhibiting significantly heightened expression of EMT markers specifically *TWIST1* and *ZEB1*. Consequently, our model presents an apt platform for conducting in vitro and preclinical investigations aimed at targeting CSC metastasis [15].

The upregulation of ABC transporter genes like *ABCB1*,* ABCC1*, and *ABCG2*, in addition to other characteristics associated with CSCs, plays a role in controlling self-renewal and multi-drug resistance in CRC cell lines [52, 53]. Our results showed an increase in *ABCB1* and *ABCC1* in 5-FU-resistant Caco2 compared to parental populations. Besides, HT-29 cells showed an increase in *ABCC1* compared to parental cells. The studies support our results on the correlation of gene expression patterns in 5-FU exposed populations. For instance, it has been shown that the overexpression of *TWIST1* heightened the resistance of CRC cells to chemotherapeutic drugs, leading to increased levels of *ABCB1* and *ABCC1* expression. Conversely, silencing *TWIST1* reversed the EMT phenotype, boosted the sensitivity of CRC cells to anticancer drugs both in vitro and in vivo, and decreased the expression of *ABCB1* and *ABCC1* [54]. Along with our observation, upregulation of ABC transporters, including ABCB1, in Doxorubicin-resistant breast cancer cells, emphasizing the conserved role of these transporters in CSC-mediated resistance [39].

The differences in gene expression alterations observed between 5-FU treatment and spheroid formation in the HT-29 and Caco2 cell lines may stem from several factors. Firstly, the nature of the methods plays a role. Spheroid formation provides a suitable microenvironment for isolating CSCs, based on the idea that CSCs can grow and form 3D structures in non-adherent, serum-free conditions [55]. CSCs have unique adhesive properties that allow them to survive and proliferate without attachment, while differentiated cancer cells typically need a substrate for growth. Removing serum from the culture medium adds stress that selectively enriches CSCs [56]. Gradual chemotherapeutic drug exposure induces natural selection, allowing only drug-resistant CSC-like cells to survive, while eliminating non-CSCs. This mirrors Darwinian selection, providing an environment that favors the survival and expansion of the CSC population [57]. In addition, chemotherapeutic drugs can induce CSC-like traits in certain cancer cells through the activation of stemness pathways, a process known as therapy-induced CSC formation or drug-induced plasticity [58, 59]. This means that while spheroid formation enriches pre-existing CSCs, chemotherapy can induce stemness features in non-CSCs.

Another key factor contributing to the differences is the distinct characteristics of the HT-29 and Caco-2 cell lines. Both are derived from human colon adenocarcinoma, but they have different differentiation potentials. HT-29 can differentiate into enterocytes, goblet cells, and mucus-secreting cells under specific conditions, whereas Caco2 primarily differentiates into enterocytes, forming a polarized monolayer with brush border microvilli [60]. Moreover, the two cell lines differ in gene expression profiles. HT-29 shows higher expression of genes related to inflammation and angiogenesis, while Caco2 has higher expression of genes involved in intestinal epithelial functions, such as nutrient absorption and barrier formation [60–62].

In our study, we observed baseline differences in several stemness and EMT genes, including *OCT-4*,* C-MYC*,* KLF4*,* SOX2*,* NANOG*,* Vimentin*,* SNAIL1*,* TWIST1*,* N-cadherin*,* E-cadherin*, and *ZEB1*. Notably, these markers were generally more highly expressed in HT-29 than in Caco2, except for *NANOG*, which was more prominent in Caco2. This aligns with our findings that neither 5-FU treatment nor spheroid formation significantly elevated *NANOG* expression in Caco2, likely because it is already highly expressed. In contrast, spheroid formation in HT-29 increased *NANOG* expression, highlighting a difference in how the two cell lines respond (data are not shown).

Another important distinction between HT-29 and Caco2 lies in their resistance to chemotherapeutic drugs. Studies have shown that HT-29 shows more resistance features compare to other human colorectal adenocarcinoma cells [63]. We showed that HT-29 cells are more resistant to 5-FU, with an IC50 of 543.3 ng/ml compared to 353.4 ng/ml for Caco2. This higher resistance may explain why HT-29 is less prone to gaining additional stemness features following drug treatment, as it likely relies on other compensatory mechanisms, such as the higher expression of ABC transporters (specifically ABCC1 and ABCG2), compared to Caco2 (data are not shown).

In order to shed light on pathways involved based on the expression pattern of genes and markers, we carried out bioinformatic analysis. Examining the shared differentially expressed genes in both the parental and 5-FU exposed subpopulations of Caco2 and HT-29 cells unveiled a notable intersection with enriched pathways documented in the KEGG database. These pathways encompassed microRNAs in cancer, pathways that control stem cells’ pluripotency and proteoglycans in cancer. The upregulated *ABCB1*,* ZEB1*, and CD44 were engaged in microRNA regulation in cancer, *SOX2 and KLF4* were engaged in pluripotency of stem cells, and *TWIST1* and CD44 contributed to proteoglycans in cancer. It has been shown that the interactions between hyaluronic acid (HA) and CD44 have demonstrated pivotal functions in drug resistance, angiogenesis, metastasis, and tumor cell survival. Moreover, TME rich in HA influences various processes that promote the self-renewal, migration, and establishment of CSCs in diverse niches. Besides, this interaction results in upregulation of *TWIST1*, leading to enhanced EMT and invasion [64]. The bioinformatic results cover pivotal CSC phenomena, including pluripotency and EMT.

It’s imperative to recognize some limitations inherent in this study. Employing only one chemotherapy agent and a restricted range of CSC markers offers a targeted yet limited perspective on the intricate dynamics within the TME. Furthermore, our research was confined solely to in vitro experimentation, potentially failing to capture the complete spectrum of in vivo conditions and the systemic impacts of chemotherapy on CSC populations. Future studies should explore functional assays, metabolic profiling, and drug response testing to confirm the therapeutic relevance of these findings.

## Conclusion

In conclusion, the 5-FU-exposure method effectively resulted in the enrichment of a subset of cells displaying CSC characteristics comparable to those CSCs isolated through spheroid culture. The observed morphological alterations and upregulation of stemness, migration, EMT-related, and ABC genes in the 5-FU resistant CRC cell populations showed the efficacy of this method for isolating CSC-like population. Our findings suggest that drug exposure methods have more priorities than spheroid formation, including better efficacy in stemness, EMT, scavenging system, and surface markers that bring it up as a candidate protocol for establishing CSCs experimental models.

## Data Availability

No datasets were generated or analysed during the current study.
