# Correction: Molecular and phenotypic characterization of 5-FU resistant colorectal cancer cells: toward enrichment of cancer stem cells

**DOI:** 10.1186/s12935-025-03891-y

**Published:** 2025-07-04

**Authors:** Amirhesam Babajani, Saeed Rahmani, Mohammad Jamal Asadi, Elmira Gheytanchi, Glavizh Adibhesami, Faezeh Vakhshiteh, Zahra Madjd

**Affiliations:** 1 Oncopathology Research Center, Iran University of Medical Sciences, Tehran, Iran; 2 Department of Computer Engineering, Sharif University of Technology, Tehran, Iran; 3 Department of Molecular Medicine, Faculty of Advanced Technologies in Medicine, Iran University of Medical Sciences, Tehran, Iran

**Correction to: Cancer Cell International (2025) 25:154**

10.1186/s12935-025-03758-2

In this article [1], the author’s name Amirhesam Babajani was incorrectly written as Amirhesam Babajnai.

Incorrect author name:

Amirhesam Babajnai

Correct author name:

Amirhesam Babajani
